# Protein requirements of hair sheep raised in warm areas: a multi-study approach

**DOI:** 10.1038/s41598-022-23199-5

**Published:** 2022-10-28

**Authors:** Caio Julio Lima Herbster, Marcilio de Sousa Mendes, Alessandra Pinto de Oliveira, Marcos Inácio Marcondes, João Paulo Pacheco Rodrigues, Luciano Pinheiro da Silva, Stefanie Alvarenga Santos, Izabelle Auxiliadora Molina de Almeida Teixeira, José Augusto Gomes Azevêdo, Elzania Sales Pereira

**Affiliations:** 1 Animal Science Department, Universidade Federal Do Ceara, 2977, Mister Hull Avenue, Fortaleza, 60356000 Brazil; 2 Department of Animal Science, Washington State University, Clark Hull, Pullman, WA 99163 USA; 3 Animal Science Institut, Universidade Federal Rural do Rio de Janeiro, Km 07, BR 465 Highway, Seropédica, 23890000 Brazil; 4 School of Veterinary Medicine and Animal Science, Universidade Federal da Bahia, 500, Adhemar de Barros Avenue, Salvador, 40170110 Brazil; 5 Department of Animal, Veterinary and Food Sciences, University of Idaho, Twin Falls, 83303-1827 USA; 6 Department of Agricultural and Environmental Science, Universidade Estadual de Santa Cruz, Km 16, Jorge Amado Highway, Ilheus, 45662900 Brazil

**Keywords:** Animal physiology, Metabolism

## Abstract

The protein requirements of hair sheep are a key factor in increasing profitability and decreasing the environmental impacts of nitrogen excretion. The objective of this study was to evaluate the protein requirements of hair sheep. A database with 382 individual records (269 intact and 113 castrated males) comprising information from 11 studies was used. The studies provided different levels of metabolisable protein intake (MPI) and of N retention, allowing the development of equations to predict the net protein (NP) and the metabolisable protein (MP) requirements. The efficiency of MP use for gain (*k*_*pg*_) was calculated using the equation of daily protein retained against daily MPI above maintenance. The efficiency of MP use for maintenance (*k*_*pm*_) was computed as the ratio between the NP for maintenance (NPm) and the MP for maintenance (MPm). The NPm (1.32 g/kg^0.75^ EBW) did not differ between sex. The *k*_*pm*_ was 0.34, and the *k*_*pg*_ was 0.25. The MPm estimated was 3.21 g/kg^0.75^ BW. Sex affects the protein requirements for gain (NPg). The protein requirements of hair sheep differ from those recommended by feeding systems for sheep. The equations provided herein may improve the optimisation of protein nutrition of sheep, thereby minimising the environmental impacts of sheep production.

## Introduction

The determination of protein requirements of hair sheep is a key step in calculating the adequate protein supply. Hair sheep has significant importance in tropical regions^1^^.^ The knowledge of nutrient requirements and efficiency of utilization of feed resources is important to optimize productivity and achieve expected performance^2,3^. In addition, it will allow food strategies and cost reduction in the formulation of diets. Nutrient requirements vary across species (NRC^4,5^ and CSIRO^6^), breeds and animal category^7^.

Accurate information regarding the protein requirements of hair sheep and the factors that affect^8^ them is essential to estimate the body protein content of growing hair sheep^9^. One of these factors is sex^7^, which may affect the tissue deposition and consequently differ in their body protein between castrated and intact males^10^. Intact males have a higher growth rate, with gain composition characterized by higher protein content^11^. The effect of sex is not reported in the protein requirements for maintenance by the current feeding systems^5,6^.

The protein requirements for male hair sheep raised in tropical area may be different from those suggested in feeding system, which were elaborated from experiments with wool animals in other conditions of temperature and climate. In addition, information regarding the protein requirements of hair sheep and the factors that affect them is essential to accomplish efficient diet formulation. Efforts have been made to determine the nutrient requirements of hair sheep, and several studies have been conducted at our institution to estimate the protein requirements for maintenance and growing. In this study we are using the meta-analytical approach to estimate the protein requirements for the maintenance and growing of intact and castrated males. Our hypothesis is that sex influences the protein requirements of male hair sheep. Therefore, the objective of this study was to determine protein requirements by using individual data in a multi-study approach.

## Results

### Metabolisable protein requirements for maintenance

**Figure 1:**
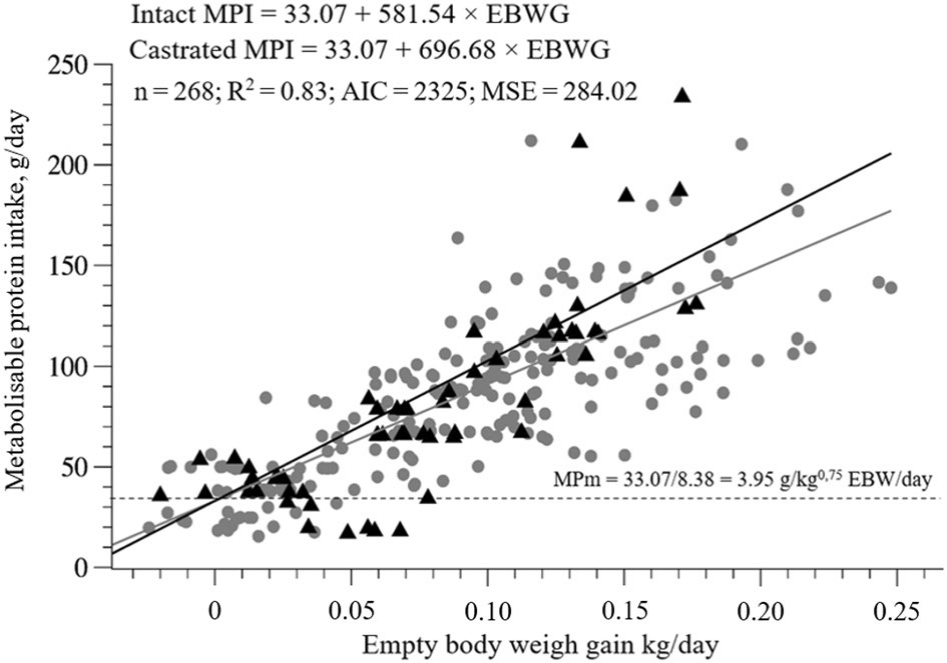
Predicted equations by the relationship between the metabolisable protein intake against empty body weight gain of hair sheep.

Sex did not influence the intercept (*P* =0.1042) of the equation of metabolisable protein intake (MPI, g/day) against empty body weight gain (EBWG, kg/day), showing no difference between intact and castrated males for metabolisable protein requirements for maintenance (MPm). When we divided the intercept (33.07) of the equation by the average metabolic empty body weight (kg^0.75^ EBW) of our database (8.38 kg), the MPm value was 3.95 g/kg^0.75^ EBW/day (Fig. 1). However, the slope of the models was influenced by sex, generating two Eqs. (1) and (2):

1 $$Intact\;MPI =33.07+581.54\times EBWG$$

2 $$Castrated\;MPI=33.07+696.68\times EBWG$$

### Net protein requirements for maintenance

**Figure 2:**
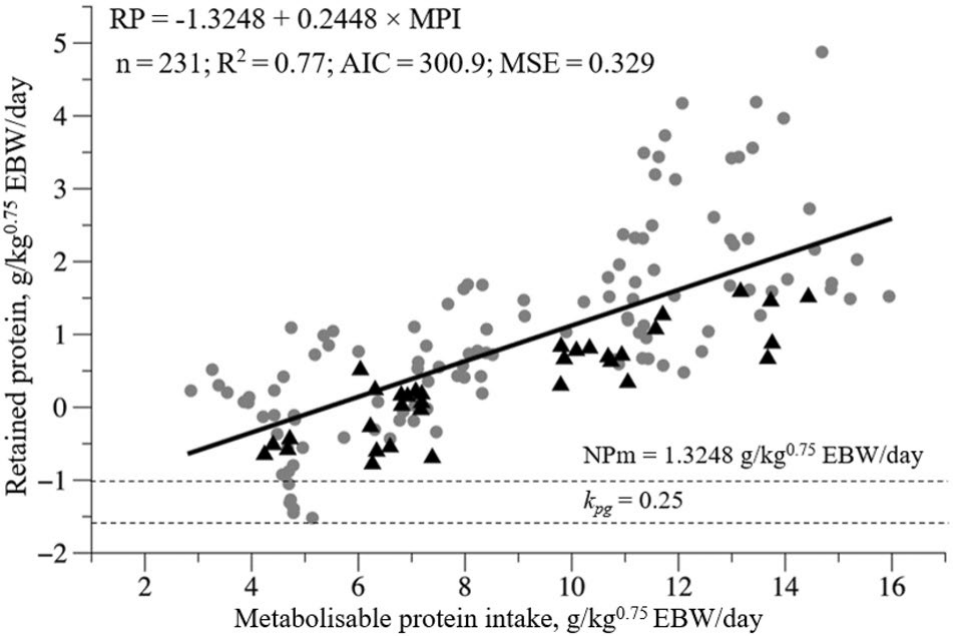
Predicted equation by the relationship between the retained protein against metabolisable protein intake of hair sheep.

Sex did not influence the net protein requirements for maintenance (NPm); thus, an equation was generated for both sex: Retained protein (RP) = − 1.3248 + 0.2448 × MPI (*P* = 0.1441) (Fig. 2). The NPm was 1.32 g/kg^0.75^ EBW/day (Fig. 2). The ratio between NPm and MPm generated a k_*pm*_ of 0.34.

### Net protein requirements for weight gain

**Figure 3:**
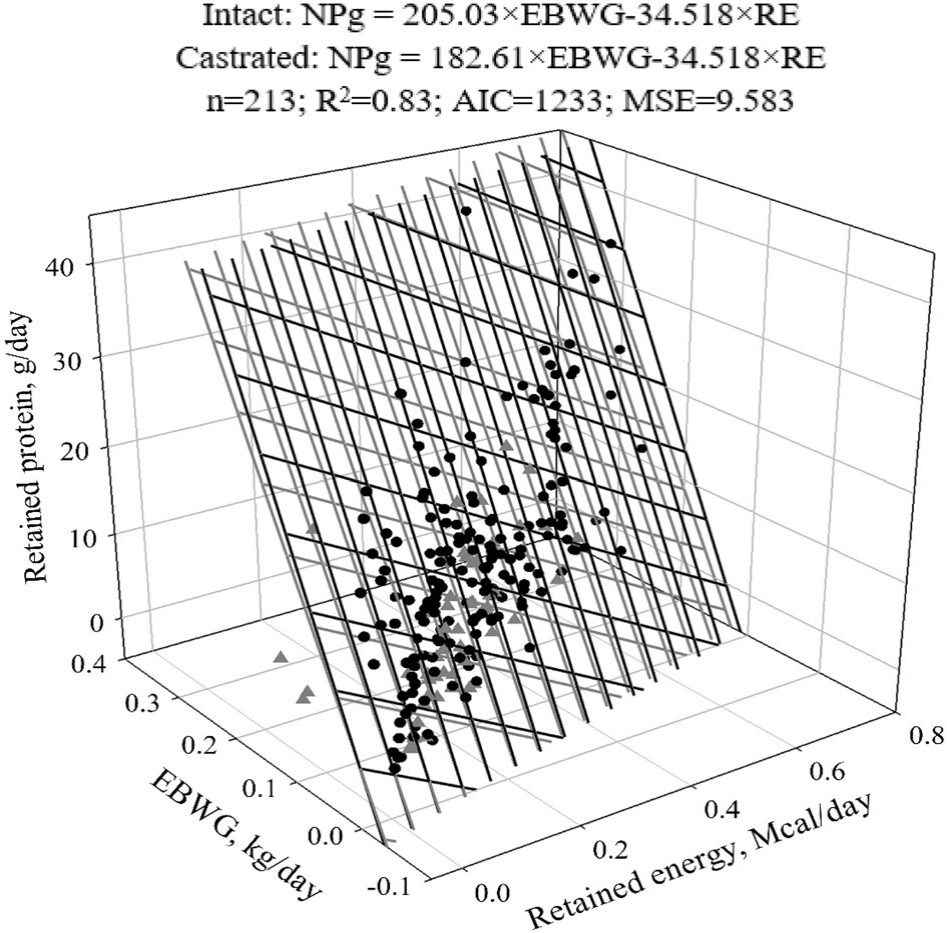
Predicted equations of net protein requirements for weight gain of intact and castrated males.

There was an effect of sex on net protein requirements for weight gain (NPg) only for the first slope of the equation (*P* = 0.0006) (Fig. 3). Therefore, two Eqs. (3) and (4) without intercepts were fitted to determine the NPg (g/day) of intact and castrated males:

3 $$Intact\;NPg = 205.03 \times EBWG {-} 34.518 \times RE$$

4 $$Castrated\;NPg =182.61\times EBWG-34.518\times RE$$

The NPg and the metabolisable protein requirements for weight gain (MPg) were higher for intact males and decreased as body weight increased (Tables 1, 2). The NPg and MPg for intact males with 30 kg BW and an ADG of 150 g/day were 14.22 and 56.89 g/day, respectively. Castrated males had lower requirements (27%) compared to intact males, showing NPg and MPg of 11.18 and 44.70 g/day, respectively. The slope of Eq. (14) corresponds to a *k*_*pg*_ of 0.25.

**Table 1:** Protein requirements for intact males.

BW (kg)	FBW (kg)	EBW (kg)	ADG (g/day)	EBWG (g/day)	NPm (g/day)	NPg (g/day)	MPm (g/day)	MPg (g/day)	TMP (g/day)
10	8.77	6.23	0.100	0.091	5.20	14.74	15.58	58.95	74.53
10	8.77	6.23	0.150	0.136	5.20	22.68	15.58	90.72	106.31
10	8.77	6.23	0.200	0.181	5.20	30.74	15.58	122.96	138.54
20	18.08	14.44	0.100	0.091	9.78	11.37	29.27	45.46	74.73
20	18.08	14.44	0.150	0.136	9.78	18.13	29.27	72.52	101.79
20	18.08	14.44	0.200	0.181	9.78	25.11	29.27	100.44	129.70
30	27.39	22.65	0.100	0.091	13.70	8.47	41.02	33.88	74.90
30	27.39	22.65	0.150	0.136	13.70	14.22	41.02	56.89	97.91
30	27.39	22.65	0.200	0.181	13.70	20.27	41.02	81.10	122.11

*BW* body weight, *FBW* fasting body weight, *EBW* empty body weight, *ADG* average daily gain, *EBWG* empty body weight gain, *NPm* net protein requirements for maintenance, *NPg* net protein requirements for weight gain, *MPm* metabolisable protein requirements for maintenance, *MPg* metabolisable protein requirements for weight gain, *TMP* total metabolisable protein.

**Table 2:** Protein requirements for castrated males.

BW (kg)	FBW (kg)	EBW (kg)	ADG (g/day)	EBWG (g/day)	NPm (g/day)	NPg (g/day)	MPm (g/day)	MPg (g/day)	TMP (g/day)
10	8.77	6.23	0.100	0.091	5.20	12.71	15.58	50.82	66.41
10	8.77	6.23	0.150	0.136	5.20	19.63	15.58	78.54	94.12
10	8.77	6.23	0.200	0.181	5.20	26.68	15.58	106.71	122.29
20	18.08	14.44	0.100	0.091	9.78	9.33	29.27	37.34	66.61
20	18.08	14.44	0.150	0.136	9.78	15.08	29.27	60.34	89.60
20	18.08	14.44	0.200	0.181	9.78	21.05	29.27	84.19	113.45
30	27.39	22.65	0.100	0.091	13.70	6.44	41.02	25.76	66.78
30	27.39	22.65	0.150	0.136	13.70	11.18	41.02	44.70	85.72
30	27.39	22.65	0.200	0.181	13.70	16.21	41.02	64.85	105.86

*BW* body weight, *FBW* fasting body weight, *EBW* empty body weight, *ADG* average daily gain, *EBWG* empty body weight gain, *NPm* net protein requirements for maintenance, *NPg* net protein requirements for weight gain, *MPm* metabolisable protein requirements for maintenance, *MPg* metabolisable protein requirements for weight gain, *TMP* total metabolisable protein.

## Discussion

We understand that nutrient requirements of hair sheep raised in the tropics differ from those of sheep raised in temperate regions. Thus, there is a need to assess the protein requirements of these animals. The adequate estimation of protein requirements is an important factor in calculating the adequate supply of this nutrient. Sex is one of the factors that influence the chemical constituents of the animal's body^12^ and, consequently, the nutritional requirements^6^. The protein requirement for growth is dependent on the content of fat-free dry matter in weight gain^7^. In our study the sex influenced the protein requirement for growth. Intact males deposit more fat-free body tissue than castrated, and therefore higher protein requirement for gain.

The metabolisable protein refers to the pool of amino acids (AA) absorbed by the animal^5,6,13,14^. The quantity and quality of AA absorbed in the intestine are essential for all vital processes in the body. The use of crude protein intake to estimate protein requirements leads to greater prediction errors^15^ as it disregards the biological value of crude protein (CP)^16^ and the efficiency of microbial crude protein (MCP) synthesis^17^.

In our study, the MPm requirement was 3.95 g/kg^0.75^ EBW/day. Converting to the BW and fasting BW (FBW) basis, the values obtained were 3.26 g/kg^0.75^ FBW/day and 3.21 g/kg^0.75^BW/day, respectively. Our value (3.21 g/kg^0.75^ BW/day) is similar to that reported by Wilkerson et al.^18^ for beef cattle (3.8 g/kg^0.75^ BW/day). For growing goats, the value of 3.07 and 3.8 g/kg^0.75^ BW/day was observed by Luo et al.^19^ and Souza et al.^20^, respectively. It is also interesting to note that the method we used to determine MPm is different from that used by the NRC^5^, in which the requirements were calculated from the sum of faecal, urinary, scurf and fibre losses. However, using the approach of Wilkerson et al.^18^, the NRC^5^ reports a MPm requirement equal to 2.51 g/kg^0.75^ BW. Thus, the use of metabolisable protein intake would be more appropriate as it includes the AA truly available in the small intestine from MCP and rumen-undegradable protein (RUP), based on animal growth rather than nitrogen balance^13,21^. Differences in MPm requirements can also be attributed to the biological value of dietary protein. Animals fed forages with a low nutritional value tend to retain low nitrogen (N) and, consequently, have high protein requirements^22^.

The estimated value of NPm was 1.32 g/kg^0.75^ EBW/day; converted to FBW and BW basis, the values obtained were 1.09 g/kg^0.75^ FBW/day and 1.07 g/kg^0.75^ BW/day, respectively. These values were close to those reported by Pereira et al.^7^ and Pereira et al.^23^, i.e., 1.30 g/kg^0.75^ FBW/day and 1.06 g/kg^0.75^ BW/day, respectively. However, the AFRC^13^ suggests 2.18 g/kg^0.75^ FBW/day as a requirement of NPm. These differences may be related to the methodologies used to estimate NPm requirements. The AFRC^13^ estimates NPm through N-free diets and intragastric N infusion, which may overestimate N excretion^6^. The NRC^5^ and CSIRO^6^ use empirical equations to estimate N excreted in faeces. The variations in the protein requirement may be related to factors such as breed, sex class, physiological status and environments factors.

Conceptually, NPg represents the amount of CP retained in the body as the animals grow^21^, being determined by genetic potential and the influence on which environmental conditions allow its expression. Among the factors that affect animal growth, nutrition stands out as it determines the supply of nutrients for tissue retention. However, this retention does not respond directly to the supply of nutrients. Protein accretion, for instance, is established up to a theoretical maximum limit after which fat deposition becomes the main component of energy retention^24^. The NPg is directly affected by body gain composition, which is considered in the model by adding the retained energy (RE)^21^. Generally, NPg values are higher for intact animals and those of late maturity^10^. Intact males deposit more fat-free body tissue than castrated, resulting in a higher protein requirement for gain^25^. In our study, the requirements were estimated at 17.29 and 9.46 g/day, 14.24 and 6.41 g/day of NPg for intact and castrated males, respectively, both with 20 and 40 kg of BW for the same rate of weight gain (150 g/day). The decrease in the NPg with increasing BW in our study is due to the reduction in muscle growth and the increase in adipose tissue development. This demonstrates that the protein stabilises as the animal approaches maturity, corroborating the approach of the NRC^21^ and BR-CORTE^16^ that chemical maturity can be achieved by stabilising protein accumulation in the EBW.

To convert NPg to MPg, we determined the efficiency of the use of metabolisable protein for gain (*k*_*pg*_*)*. The efficiency of MP use represents the amount of absorbed AA used to replace protein losses by the body, tissue protein retention and milk protein secretion. The AA profile of dietary feedstuffs has been mentioned as the main factor affecting the efficiency^26^. Our results agree with this affirmation, with a *k*_*pg*_ value of 0.25. High forage ratios in diets can increase protein requirements^22^. Another approach is that warm areas associated with high humidity may induce specificities in food characteristics as well as in animals. High temperatures in the tropics are correlated with increased AA requirements in the growth phase^27^, possibly due to the N recycling required for tissue regeneration.

Committees adopt fixed values to express the efficiency of MP use for maintenance, such as 0.75 for ARC^12^, 0.70 for CSIRO^6^, 1.0 for AFRC^13,28^ and 0.67 for NRC^5^. For growth, efficiencies of 0.59 have been reported for AFRC^13^ and 0.70 for both CSIRO^6^ and NRC^5^. Our study suggests *k*_*pm*_ and *k*_*pg*_ values of 0.34 and 0.25, respectively. These values are compatible with the idea that the efficiency of metabolisable protein use is influenced by the energy supply, which is possibly associated with the reduction in the use of AA for hepatic gluconeogenesis as the energy intake is high. However, the efficiency of using a balanced AA mixture is also a characteristic of the animal^26^ and varies depending on factors such as breed and physiological stage. The uncertainty of the real efficiency for gain and maintenance may increase the variability among the recommendations.

In conclusion, we suggest that there is no evidence that sex class affects the protein requirements for maintenance. However, it influences the net protein requirements for gain. The generated equations may improve the accuracy of protein requirement values adopted and help nutritionists optimise protein levels in hair sheep diets, thereby minimising the environmental impacts.

## Methods

### Ethical considerations

Approval by an ethics committee in the use of animals was not necessary in this study since data were collected from previously published sources.

### Model proposal

Only experiments conducted with hair sheep or crosses raised in tropical regions of Brazil that reported individual information of the following quantitative data: BW, EBW, average daily gain (ADG), EBW gain (EBWG), total digestible nutrient intake (TDNI), crude protein intake (CPI), and body protein (BPC) and fat (BFC) contents. The studies contained information on individual animals fed at least two levels above maintenance and at maintenance levels, based on a comparative slaughter methodology.

The database consisted of 11 experimental studies (Nascimento Junior^29^; Silva et al.^30^; Pereira^31^; Costa et al.^32^; Regadas Filho et al.^9^; Oliveira et al.^33^; Rodrigues et al.^34^; Pereira et al.^35^; Pereira et al.^7^; Pereira et al.^23^, and Mendes et al.^36^), comprising a total of 382 animals. Of these, 74 animals belonged to the reference group and 308 to the experimental groups, with two sex classes: intact (n = 269) and castrated (n = 113) males. The dietetic crude protein (CP) and metabolisable energy (ME) ranged from 47 to 236 g/kg of dry matter (DM) and from 0.9 to 3.4 Mcal/kg DM, respectively; the main feeding system was the feedlot (Table 3). The Nascimento Junior^29^ study was not included to estimate the protein requirements for maintenance due to the lack of intake information (TDNI and CPI).

**Table 3:** Characteristics of studies included in the database for estimating the protein requirements.

Studies	n	Breed	Sex	Feeding System	CP (g/kg DM)	ME (g/kg DM)
Nascimento Júnior^29^	30	Dorper and Santa Ines	CM	Pasture	–	–
Silva et al.^30^	32	Santa Ines	CM	Pasture	50–224	1.6–2.3
Pereira^31^	30	Santa Ines	IM	Feedlot	129–174	2.0–2.1
Costa et al.^32^	47	Morada Nova	IM	Feedlot	47–181	0.9–3.1
Regadas Filho et al.^9^	23	Santa Ines	IM	Feedlot	143–230	2.1–2.7
Oliveira et al.^33^	34	Santa Ines	IM	Feedlot	104–205	1.7–3.4
Rodrigues et al.^34^	36	Non-descript breed	IM and CM	Feedlot	204–230	1.7–2.5
Pereira et al.^35^	47	Brazilian Somali	IM	Feedlot	88–202	0.9–2.9
Pereira et al.^7^	37	Santa Ines	IM and CM	Feedlot	154–236	1.6–2.6
Pereira et al.^23^	31	Morada Nova	IM and CM	Feedlot	169–173	2.1–2.7
Mendes et al.^36^	35	½ Dorper x ½ Santa Ines	IM	Feedlot	139–150	2.0–2.5

*n* number of experimental units, *DM* dry matter, *CP* crude protein, *ME* metabolisable energy, *IM* intact male, *CM* castrated male.

### Slaughter, chemical analysis and body composition

All studies used the methodology of comparative slaughter. After slaughter, the body components were analysed for DM content (AOAC^37^; method 930.15); the fat content was determined by ether extraction (EE) using a Soxhlet apparatus for 12 h (AOAC^37^; method 920.39) and CP (AOAC^37^; method 984.13). Overall, measures of intake, digestibility and calculations of ME intake, retained energy (RE) and retained protein (RP) were similar across the studies, and details can be accessed directly in the original publications. The EBW, BPC and BFC of the reference animals, slaughtered at the beginning of the experiments, were used to estimate the initial EBW, BPC and BFC of the experimental animals, individually. The body energy content (BEC) of the animals of each study was calculated by the equation recommended by the ARC^12^:

5 $$\mathrm{BEC}=\left(\mathrm{BPC}\times 5.6405\right)+(\mathrm{BFC}\times 9.3929)$$

where BEC is the body energy content (Mcal/day), BPC is the body protein content (kg), BFC is the body fat content (kg). The RP and RE were estimated by the difference between the final BPC and BFC and the initial BPC and BFC of each study, respectively. The descriptive statistics of the variables used to fit the models are shown in Table 4.

**Table 4:** Description of variables used to estimate of the protein requirements of hair sheep.

Item	n	Mean	Standard deviation	Minimum	Maximum
BWi (kg)	308	17.61	6.97	9.00	39.60
BWa (kg)	308	22.12	6.95	10.07	43.47
BWf (kg)	382	24.83	8.67	7.07	54.40
EBWi (kg)	308	13.44	5.89	5.94	31.54
EBWa (kg)	308	17.19	5.82	7.07	34.09
EBWf (kg)	382	19.45	7.27	6.34	41.66
EBW^0.75^ (kg)	308	8.36	2.08	4.34	14.11
ADG (kg/day)	308	0.101	0.073	− 0.101	0.366
EBWG (kg/day)	308	0.084	0.066	− 0.147	0.318
MPI (g/day)	283	82.21	41.44	15.49	233.73
RE (Mcal/kg^0.75^ EBW/day)	273	0.0286	0.0173	0.0001	0.0795

*n* number of experimental units, *BWi* initial body weight, *BWa* average body weight, *BWf* final body weight, *EBWi* initial empty body weight, *EBWa* average empty body weight, *EBWf* inicial empty body weight, *ADG* average daily gain, *EBWG* empty body weight gain, *MPI* metabolisable protein intake, *RE* retained energy.

### BW and body gain adjustments

Fasting body weight, empty body weight and empty body weight gain were estimated according to equations recommended by Herbster et al.^38^:

6 $$\mathrm{FBW}=-0.5470+0.9313\times \mathrm{BW}$$

7 $$\mathrm{EBW}=-1.4944+ 0.8816\times \mathrm{FBW}$$

8 $$\mathrm{EBWG}=0.906\times \mathrm{ADG}$$

where BW is the body weight (kg), FBW is the estimated fasting body weight (kg), EBW is the estimated empty body weight (kg), EBWG is the estimated empty body weight gain (kg/day), ADG is the average daily gain (kg/day). The factors 1.23 (BW/EBW) and 1.21 (FBW/EBW) were used to convert the requirements expressed in g/kg EBW into g/kg BW and g/kg FBW, respectively.

### Metabolisable protein intake

The MCP synthesis was estimated using the equation recommended by Santos et al.^39^:

9 $$\mathrm{MCP}=12.7311+59.2956\times \mathrm{TDNI}$$

where MCP is the estimated microbial crude protein synthesis (g/day) and TDNI is the total digestible nutrient intake calculated for each study (kg/day). Posteriorly, the rumen degradable protein (RDP) was considered equal to MCP. To estimate the truly digestible microbial crude protein, the following equation was used:

10 $$\mathrm{tdMCP}=\mathrm{RDP}\times 0.64$$

where tdMCP is the truly digestible microbial crude protein (g/day), RDP is the estimated rumen-degradable protein (g/day), 0.64 is the value considering that the MCP is constituted of 80% amino acids with an intestinal digestibility of 80%^21^. The RUP intake was calculated as the difference between CP intake and RDP. Therefore, the digestible rumen undegradable protein was obtained from the following equation:

11 $$\mathrm{dRUP }=\mathrm{ RUP }\times 0.80$$

where 0.80 refers to the 80% digestibility of RUP in the small intestine^21^. Thus, the metabolisable protein intake (MPI) was calculated as the sum of tdMCP and dRUP.

### Metabolisable protein requirements for maintenance

The metabolisable protein requirement for maintenance (MPm, g/kg^0.75^ EBW/day) was estimated from the adaptation of equations provided by Wilkerson et al.^18^ and the NRC^21^. Initially, a linear regression of MPI against the EBWG of the animals was fitted:

12 $$\mathrm{MPI}={\upbeta }_{0}+{\upbeta }_{1}\times \mathrm{EBWG}$$

where MPI corresponds to the metabolisable protein intake (g/day), EBWG is the empty body weight gain (kg/day) and β_0_ and β_1_ are the linear regression coefficients. Posteriorly, the intercept (β_0_) of the adjusted model was divided by the general average metabolic EBW of the animals, and this result was assumed as the MPm (g/kg^0.75^ EBW/day):

13 $$\mathrm{MPm}= \frac{{\upbeta }_{0}}{{\mathrm{EBW}}^{0.75}}$$

### Net protein requirements for maintenance

To estimate the net protein requirement for maintenance (NPm, g/kg^0.75^ EBW/day), a linear regression of the RP against the MPI was fitted, according to the following equation:

14 $$\mathrm{RP}={\upbeta }_{0}+{\upbeta }_{1}\times \mathrm{MPI}$$

where RP is the retained protein (g/kg^0.75^ EBW/day), MPI is the metabolisable protein intake (g/kg^0.75^ EBW/day), β_0_ was considered as NPm, and β_1_ was the efficiency of metabolisable protein use for weight gain (*k*_*pg*_). The efficiency of metabolisable protein use for maintenance (*k*_*pm*_) was obtained as NPm/MPm.

### Net protein requirements for weight gain

To estimate net protein, the requirement for weight gain (NPg), a regression between RP against EBWG and RE was fitted. This method considers that animal performance and body gain composition are correlated with the proportion of the energy retained in the gain^21^:

15 $$\mathrm{NPg}={\upbeta }_{0}+{\upbeta }_{1}\times \mathrm{EBWG}+{\upbeta }_{2}\times \mathrm{RE}$$

where NPg is the net protein requirement for weight gain (g/day), EBWG is the empty body weight gain (kg/day), RE is the retained energy (Mcal/day), β_0_, β_1_ and β_2_ are the linear regression coefficients.

### Statistical analysis

A linear mixed model was used to estimate and test parameters and effects in this study. As the dataset comprises different individual studies, we used a meta-analysis approach incorporating the study effect as a random effect^40^. The inclusion of the study effect was also tested for each model slope and intercept. Fixed effects of sex classes on model parameters were tested, and when the differences were significant (*P* < 0.05), a unique equation for all sex classes was used. Normality and dispersion of residuals were checked, and we considered as influential points the records with studentised residuals greater than 2.5 and/or Cook’s distance greater than 1^41–44^. We tested three covariance structures in this study, using first an unstructured covariance, and with no convergence and/or no significance of covariance (*P* < 0.05), variance components (VC) and compound symmetry (CS) structures were tested and chosen based on the corrected AIC value. Statistical analysis was performed using, respectively for linear mixed and nonlinear mixed models, the MIXED and NLMIXED procedures of SAS (SAS Institute Inc).

## Data Availability

The datasets analyzed during the current study are available from the corresponding author on reasonable request.
